# The Translation and Commercialisation of Biomarkers for Cardiovascular Disease—A Review

**DOI:** 10.3389/fcvm.2022.897106

**Published:** 2022-06-02

**Authors:** Soloman Saleh, Jacob George, Katharine A. Kott, Peter J. Meikle, Gemma A. Figtree

**Affiliations:** ^1^Cardiothoracic and Vascular Health, Kolling Institute of Medical Research, Sydney, NSW, Australia; ^2^Faculty of Medicine and Health, University of Sydney, Sydney, NSW, Australia; ^3^Department of Cardiology, Royal North Shore Hospital, Northern Sydney Local Health District, Sydney, NSW, Australia; ^4^Baker Heart and Diabetes Institute, Melbourne, VIC, Australia; ^5^Charles Perkins Centre, University of Sydney, Sydney, NSW, Australia

**Keywords:** cardiovascular disease, biomarker, patents, personalized medicine, translational medicine, commercialization, risk stratification

## Abstract

As a leading cause of mortality and morbidity worldwide, cardiovascular disease and its diagnosis, quantification, and stratification remain significant health issues. Increasingly, patients present with cardiovascular disease in the absence of known risk factors, suggesting the presence of yet unrecognized pathological processes and disease predispositions. Fortunately, a host of emerging cardiovascular biomarkers characterizing and quantifying ischaemic heart disease have shown great promise in both laboratory settings and clinical trials. These have demonstrated improved predictive value additional to widely accepted biomarkers as well as providing insight into molecular phenotypes beneath the broad umbrella of cardiovascular disease that may allow for further personalized treatment regimens. However, the process of translation into clinical practice – particularly navigating the legal and commercial landscape – poses a number of challenges. Practical and legal barriers to the biomarker translational pipeline must be further considered to develop strategies to bring novel biomarkers into the clinical sphere and apply these advances at the patient bedside. Here we review the progress of emerging biomarkers in the cardiovascular space, with particular focus on those relevant to the unmet needs in ischaemic heart disease.

## Introduction

The burden of cardiovascular disease (CVD) has steadily increased over the past 30 years, contributing to significant morbidity and mortality (1). Often diagnosis occurs late in the disease process when considerable irreversible damage has already occurred during the subclinical phase (2). In the case of coronary artery disease (CAD), physicians and individual community members are limited to measuring risk factors that increase the probability of developing atherosclerotic disease and cardiovascular events such as cholesterol levels, blood pressure, and diabetes. However, up to 27% of first time heart attack patients have no standard modifiable risk factors (3). Furthermore, many individuals with intermediate to high risk have substantial variation in their susceptibility to disease (4–6). Thus, a biomarker of atherosclerosis itself, and particularly the more vulnerable plaque features would be the “holy grail” for tackling CAD. As our understanding of the complexity of heart disease increases, our ability to detect and quantify those complexities needs to increase accordingly. More and more we are recognizing CVD as multiple interrelated processes with a spectrum of phenotypes, rather than a simple linear progression of disease (7). Additionally, if it were to relate directly to the burden of disease in a given patient, the biomarker could guide individualized therapeutic interventions, furthering the transition toward personalized medicine. Many current biomarkers have been discovered due to their pathophysiological significance in CVD, however further discovery work has been augmented by the use of omics, unbiased analyses, and other high throughput technologies (8, 9). This manuscript gives an overview of emerging cardiac biomarkers and biomarker panels measurable in simple peripheral blood samples and at various stages of the translational pipeline, and considers the commercialization landscape that shapes this. Following this is a discussion of the feasibility, challenges, and opportunities present in the contemporary cardiovascular biomarker field, particularly in relation to subclinical disease detection and the commercial considerations surrounding regulation and patenting. The latter has particular importance to understanding pathways accelerating patient access to the potential benefits of novel biomarkers.

## Emerging Biomarkers And Biomarker Panels For Diagnosis, Prognosis, And Quantification Of Cardiovascular Disease

**Figure 1:**
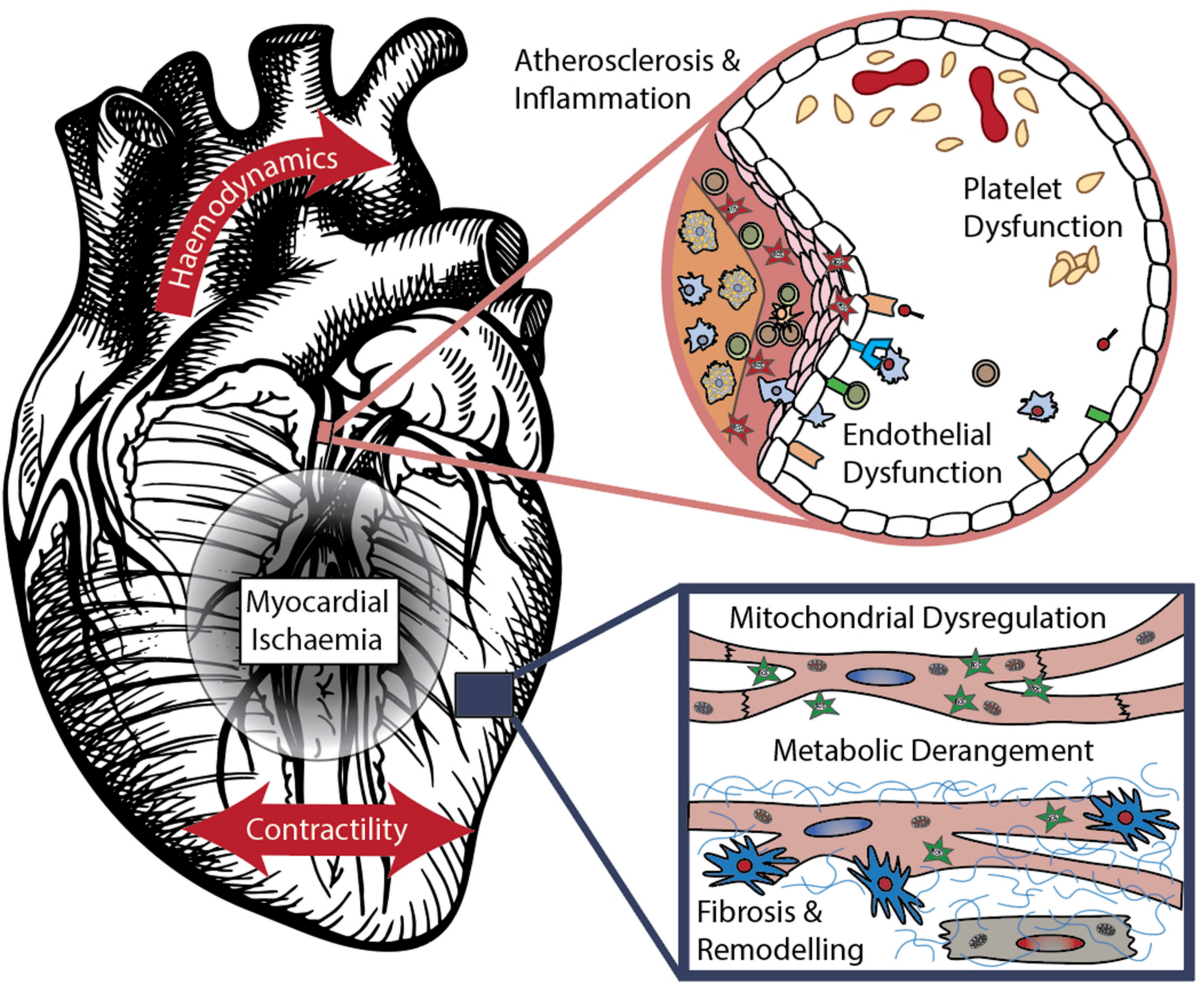
Mechanisms of cardiovascular disease – components modified from (10) with permission.

In this section we aim to summarize emerging cardiovascular biomarkers, their discovery, validation and commercialization journey. These have been grouped by their associated pathophysiological processes – atherosclerosis and platelet dysfunction, myocardial ischaemia, fibrosis and remodeling, deranged hemodynamics and contractility, and cardiometabolic disease – visualized in Figure 1. The interplay between these categories is substantial, and the exact pathophysiological processes of many biomarkers are still being elucidated. Recent patents held in these areas are summarized in Table 1. In some advanced areas, panels of multiple biomarkers have been used to compute scores focused on guiding specific treatment decisions, as further detailed in Section Biomarker Panels for Guiding Therapy.

**Table 1:** Summary of recently patented cardiovascular biomarkers, associated mechanisms and claims.

**Marker**	**Class**	**Role in health/disease**	**Measured outcome**	**Stage**	**Patent number**	**Patent description**	**Summary of patent claims**
**Atherosclerosis, plaque burden, and platelet dysfunction**							
**MMP-9** (11)	Endopeptidase	Cleaves extracellular matrix proteins as part of cardiac remodelling	Predicts plaque stability compared to ultrasound and FDG-PET	Prospective observational studies	US7319017B2	MMP-9 concentration in post-MI subjects stratifies risk of heart failure	1. Method of detection 2. Decision tool (risk calculator of 2 year heart failure risk)
**Adiponectin** (12, 13)	Peptide hormone	Has a protective cardiometabolic effect and inhibits atheroma formation	Negatively correlates with coronary artery calcification and metabolic syndrome	Prospective observational study	EP2302395B1 (A) US20100130404A1 (B)	Concentrations of biomarker panels including adiponectin predict (A) arteriovascular disease risk and (B) cardiodiabetes	1. Method of detection (A,B) 2. Comparison to reference values to predict arteriovascular risk (A) 3. Decision tool and treatment matrix for cardiodiabetes (B)
**P-selectin** (14, 15)	Protein	Pro-inflammatory cytokine Platelet aggregation Increases issue factor	Predicts CVD in healthy populations Stratifies ACS, stable angina, healthy controls	Prospective observational (case-control) studies	US7358055B2	Differentiation of myocardial ischaemia and infarction by using additional biomarker assays that include P-selectin	1. Method of detection 2. Comparison to reference values to differentiate ischaemia and infarction
**sLOX-1 and LOX-1 ligand containing ApoB (LAB)** (16)	Protein	OxLDL receptor, triggers atherogenic pathways, contributes to inflammation and fibrosis	Complexity of coronary lesions, number of vessels implicated, stability of disease. Predict cardiac or cerebral infarction	Prospective observational study	JP2013257343A	sLOX-1 and LAB concentration assist in determining cardiovascular disorder risk	1. Method of detection 2. Using sLOX-1 and LAB levels to determine cardiovascular disorder risk
**Myocardial ischaemia**							
**MIF** (17)	Protein	Pro-inflammatory cytokine released in response to myocardial ischaemia	Early marker of infarct size Predicts EF and ventricular volumes on follow up CMR	Mixed retrospective and prospective observational study	US20140234861 (A) US20200264196A1 (B) US7445886B2(C)	MIF levels can predict CV risk (C) and diagnose, prognose and treat ACS (A, B)	1. Method of detection (A, B, C) 2. Using MIF to diagnose, prognose and treat ACS (A, B) 3. Using MIF in conjunction with other tests to risk stratify and treat individuals with high CV risk (C)
**miR-137 and miR-106b-5p** (18)	Micro RNA	Nourin-dependent miR with regulatory role in cell development and apoptosis	Stratifies between normal, positive cardiac stress test, and infarction	Prospective discovery study	WO2020148589	Nourin dependent micro RNA's can enable early diagnosis of cardiac ischaemia	1. Method of detection 2. Using detection of said micro RNA's for early diagnosis of cardiac ischaemia to guide treatment
**Fibrosis and remodeling**							
**Osteopontin** (19)	Phosphorylated glycoprotein	Potential messenger role in inflammatory remodeling response	Correlation with HFrEF diagnosis and disease severity. Predicts 4-year mortality in HFrEF	Retrospective observational study	US20100267062A1	Osteopontin levels predict heart failure risk and assist in diagnosis and prognosis	1. Method of detection 2. Using osteopontin levels in conjunction with other diagnostics to determine risk, diagnosis and prognosis of heart failure
**Galectin-3** (20)	Protein	Macrophage derived pro-fibrotic agent	Predicts change in LVEDV	Prospective observational study	US20060257946A1 (A) ES2377012T3 (B)	Galectin-3 levels assist in classify cases of ischaemic heart disease (A) and determining heart failure risk (B)	1. Method of detection (A, B) 2. Using Galectin-3 levels to classify ischaemic heart diseases status. (A) 3. Detection of heart failure using Galectin-3 (B) 4. Pharmaceuticals containing galectin-3 for heart failure treatment (B)
**Phenylalanine** (21)	Amino acid	Cardiac fibrosis and senescence	Predicts 1 year survival after MI	Prospective observational study	WO2021009091	Phenylalanine levels predict cardiac dysfunction	1. Method of detection 2. Using phenylalanine levels to predict cardiac dysfunction 3. Assessing therapeutic effectiveness by monitoring changes in phenylalanine levels
**Phosphorylated cardiac troponin** (22)	Post-translational modification	May accompany limited contractile function in heart failure patients	LV remodeling on echocardiography at 1 year post-infarct	Prospective observational study	Troponin T: US20120088259A1 (A) Troponin I: EP2536760A2 (B)	Phosphorylated troponin T can detect LV remodeling and heart failure risk (A). Phosphorylated troponin I can predict heart failure risk (B)	1. Method of detection (A, B) 2. Using phosphorylated troponin T to detect LV remodeling and heart failure risk (A) 3. Using phosphorylated troponin I to determine heart failure risk and thus guide prevention and treatment (B)
**Nitrated cardiac troponin**	Post-translational modification	Unknown	Purportedly predicts myocardial ischaemia with or without infarction	Patent only, no apparent other publications	US10175250B2	Nitrated troponin I levels assist in diagnosis, prognosis and treatment of cardiac ischaemia	1. Method of detection 2. Using nitrated cardiac troponin I levels to diagnose, prognose and guide treatment of cardiac ischaemia
**Hemodynamics and myocardial contractility**							
**MICRA** (23)	Circular RNA	Unknown, likely transcriptional modulator	Levels of MIRCA detected post myocardial reperfusion correlate with ejection fraction at 4 months	Prospective observational study	US10704100B2	Circular RNA's predict heart failure risk in post MI patients	1. Method of detection 2. Using circular RNA expression in post MI patients to determine heart failure risk 3. Assessing pharmaceutical effectiveness through monitoring circular RNA expression
**Leukocyte transcriptome panel** (24)	Gene	Various, encode for membrane proteins, mitochondrial components, and transcription factors	Screening tool for ALVD	Prospective discovery study	US20130310275A1	Gene expression of a biomarker panel can diagnose ALVD	1. Method of detection 2. Using gene expression of a biomarker panel to diagnose ALVD
**Angiopoietin-2** (25)	Protein	High levels lead to endothelial and vascular instability through inhibition of angiopoietin-1	Differentiate HF from other causes of dyspnoea Predict development of HF	Retrospective observational study	US11079394B2	A biomarker panel including angiopoietin-2 predicts risk of CV events	1. Method of detection 2. A computer algorithm to enable CV risk assessment over 1–5 years
**Thrombospondin-2** (25)	Protein	Involved in mediating cellular interactions	Differentiate HF from other causes of dyspnoea Predict development of HF	Retrospective observational study	US11079394B2 (A) ES2377012T3 (B)	A biomarker panel including thrombospondin 2 predicts risk of CV events (A). Levels of thrombospondin 2 predicts risk of heart failure	1. Method of detection (A, B) 2. A computer algorithm to enable CV risk assessment over one to 5 years (A) 3. Detection of heart failure using thorombospondin 2 (B) 4. Pharmaceuticals containing thrombospondin 2 for heart failure treatment (B)
**IGFBP2** (26)	Protein	Key downstream mediator of the GH/IGF-1 pathway.	Predict mortality or significant cardiac intervention in HF patients	Prospective observational study	US20150219669A1	IGFBP2 levels assist in classifying heart failure risk.	1. Method of detection 2. Using IGFBP2 levels to determine risk of heart failure
**Metabolic derangement and mitochondrial dysregulation**							
**Lipidomic panel** (27)	Lipids	Various/not fully known. Known roles in inflammation and oxidative stress	Classifies coronary disease burden and stability	Prospective discovery study	WO2011063470	Lipidomic panel assists in the diagnosis, prognosis and risk stratification of heart disease	1. Method of detection 2. Using lipid analytes to diagnose, prognose and risk stratify heart disease
**LIPCAR** (28)	Long non-coding RNA	Unknown	Predicts HF mortality and Post-MI LV remodeling	Prospective observational studies	WO2015140224	Long non coding RNA predicts chronic heart failure mortality and post MI cardiac remodeling	1. Method of detection 2. Using expression to predict heart failure mortality 3. Using expression to predict future remodeling
**mitomiR panel** (29)	Micro RNA	Regulation of mitochondrial transcription including apoptosis, calcium homeostasis, and energy metabolism	Differentiated acute HF from acute exacerbation of COPD	Prospective discovery study	WO2016133395A1	Circulating micro RNA's can act as a marker of acute heart failure risk, presence or progression	1. Method of detection 2. Micro RNA combinations to reflect heart failure risk, presence or progression 3. Determining pharmaceutical effectiveness in heart failure through assessing changes in micro RNA expression
**MAA adducts** (30)	Breakdown product of lipid peroxidation	Proinflammatory and cytotoxic effects. May also have an impact on protein modulation	Different antibody isotypes present to MAA adducts correlate with different CAD states	Prospective observational studies	US20180313826A1 (A) US10591468B2 (B)	Antibodies to MAA adducts determine coronary artery disease risk, progression (A) and can guide treatment (A, B)	1. Method of detection (A, B) 2. Using antibodies to MAA adducts to determine CVD risk and monitor atherosclerosis progression (A) 3. Using relative antibody levels to guide treatment (A, B)
**Homoarginine** (31)	Amino acid	Possible protective cardiometabolic effect by promoting nitric oxide production	Negatively correlated to cardiovascular and all-cause mortality and of endothelial dysfunction. Possibly conflicting data	Prospective observational studies	US20130143240	Homoarginine concentration predicts risk of mortality from stroke and cardiac causes	1. Method of detection 2. Comparison to reference values to determine if homoarginine replacement is required 3. Homoarginine supplementation products
**Panels guiding therapy**							
**Aspirin response signature** (32)	RNAs	Various/unknown	Quantify effect of aspirin therapy or platelet function	Prospective discovery study	WO2014039859	Platelet function biomarkers can aid decisions regarding anti-platelet therapy	1. Method of detection 2. Using RNAs to diagnose, prognose or risk stratify CV disease. 3. Using RNA expression to determine candidates who would benefit from antiplatelet therapy 4. Using RNA expression to select and titrate anti-platelet agents
**Antihypertensive panel** (33)	Proteins	naturiesis/diuresis, pro-fibrotic, pro-remodeling	Predict outcomes of particular antihypertensive regimens	Prospective observational studies	US20150268251	A biomarker panel which identifies patients with heart failure that would benefit from pharmaceutical intervention	1. Method of detection 2. Using biomarker levels to determine individuals at risk of heart failure. 3. Biomarker analyser units to determine if individuals would benefit from pharmaceutical intervention (e.g., beta blockers)
**CRT** (34)	Proteins	Inflammatory markers and regulator of extracellular matrix	Predict likelihood of successful CRT therapy	Prospective discovery study	EP2809393	A biomarker panel which predicts a subjects response to CRT	1. Method of detection 2. Using biomarker levels to predict CRT response

### Biomarkers of Atherosclerosis, Plaque Burden, and Platelet Dysfunction

Approximately three quarters of acute coronary events are linked to atherosclerotic plaque rupture (35). Despite this, assessing individualized risk of rupture remains a challenge. Myriad radiographic and percutaneous methods exist to quantify plaque burden, each with its own limitations (36–38). Blood biomarkers quantifying degree of atherosclerosis, plaque stability, and deranged platelet function have been proposed that may give a more detailed and personalized assessment of occlusive risk without requiring invasive procedures, or better identify those who would benefit from these procedures.

#### MMP-9

The extracellular matrix proteinase, matrix metalloproteinase-9 (MMP-9), is overexpressed in stressed endothelium and is a known contributor to plaque architecture and composition (39). MMP-9 is also correlated with vascular endothelial growth factor (VEGF), a neovascularisation stimulus that further destabilizes plaque. Virtual histology-intravascular ultrasound is an invasive technique using radiofrequency backscatter patterns on an intravascular probe to measure coronary plaque burden, composition, and vulnerability (40). Elevated levels of MMP-9 are independent predictors of unstable coronary plaque; in fact, virtual histology-intravascular ultrasound during coronary angiography in 32 patients with stable coronary disease found unstable coronary plaque properties, including increased necrotic core volume and fibro-fatty content, in patients with more elevated levels of MMP-9 (11). Circulating levels of MMP-9 have also been correlated with femoral intima-media thickness, though there were mixed results regarding carotid plaques (35, 41).

In a cohort of 343 ACS patients requiring ICU admission, MMP-9 levels predicted short-term mortality while both its levels and rate of change during recovery predicted likelihood of a second major cardiac event over a 6 year follow up period (42). Likewise, in a cohort of 866 middle-aged participants apparently free of coronary disease, baseline plasma MMP-9 was associated with first-time coronary disease over an 8 year follow up, independent of known risk factors, high sensitivity CRP (hsCRP), and IL-6 (43).

#### Adiponectin

Adiponectin is a peptide hormone derived from adipose tissue that has protective cardiometabolic effects (44). This is achieved through various mechanisms including promoting insulin sensitivity and modulating lipid and glucose metabolism (45). A cross-sectional study of 284 patients found lower serum adiponectin levels were associated with metabolic syndrome status, even when confounding factors (e.g., serum lipid and insulin levels) were controlled (12). This relationship is significant given the predictive value of metabolic syndrome in the development of arteriovascular events (46).

Serum adiponectin levels are thought to also have a direct limiting effect on atherosclerosis by inhibiting key processes in atheroma formation. This includes improving endothelial cell function and suppressing smooth muscle proliferation and foam cell formation (45). A case-control study of 306 patients (101 cases, 205 controls) supports these properties as lower serum adiponectin levels were found to be associated with the progression of coronary artery calcification (13).

#### P-selectin

P-selectin is a cell-surface adhesion molecule expressed on endothelium, platelets, and leukocytes, and has been implicated in plaque development (47). In particular, platelet release of P-selectin complexes with P-selectin glycoprotein ligand-1 (PSGL-1) on leukocytes (48) activates pro-thrombotic pathways, including upregulation of tissue factor and stabilization of platelet-platelet interactions required for aggregation. In a cohort of 345 apparently healthy women, participants with elevated soluble P-selectin were at a 2.2-fold higher risk of having a CVD event than age- and sex-matched counterparts at 3.5 year follow up, independent of other risk factors (14). Similarly, in a cohort of 142 patients under 55 years old with known CAD, platelet P-selectin levels were significantly higher in ACS than stable angina, which in turn were higher than healthy controls (15).

#### Soluble LOX-1

Lectin-like oxidized low-density lipoprotein receptor 1 (LOX-1) is physiologically expressed in low amounts on endothelial cells, and sits upstream of several matrix metalloproteinases and pro-inflammatory pathways implicated in atherosclerosis (49). Receptor levels markedly increase in states of inflammation, with expression also spreading to smooth muscle and macrophages. Further, inflammatory stimuli such as TNF-α, CRP, and various interleukins trigger shedding of LOX-1 into a soluble form (sLOX-1) which has been used as a marker for a number of cardiac and vascular endpoints. sLOX-1 has been correlated with complexity of coronary lesions, number of vessels with disease, and is elevated in ACS compared to stable coronary disease (16).

#### In Summary: Future of Atherosclerotic Markers in Clinical Practice

The prospect of developing an atherosclerotic stability marker or series of markers detectable in peripheral blood may open the door to applying risk stratification or population-based screening estimating risk of plaque rupture or other myocardial event. It remains to be investigated whether these markers used in combination may give additive insight into predictability of short-term or long-term plaque rupture.

### Biomarkers of Myocardial Ischaemia

In addition to its key role in the development of atherosclerosis and vascular dysfunction, inflammation is also seen early in response to myocardial ischaemia. Crucially, these states may be present prior to ACS or within the first minutes from onset, making inflammatory mediators particularly promising cardiovascular biomarkers for detection of acute cardiac events (50).

#### MIF

Macrophage migration inhibitory factor (MIF) is an inflammatory cytokine involved in the early myocardial response to ischaemia (51). In a mouse infarct model, MIF levels were elevated at the earliest recorded point – within 15 min of occlusion and prior to evidence of infarction – and remained elevated at the 60 min mark. In a human study of 374 ST-elevation myocardial infarction (STEMI) patients, of all biomarkers measured on admission including troponins, only MIF was correlated with infarct size, ejection fraction, and ventricular volumes on cardiac MRI at the 3-day and 3-month mark (17).

#### Nourin-Dependent miRNAs

Nourin is a potent chemoattractant peptide released by ischaemic myocardium within 5 min of onset (52). In a cohort of patients being investigated for inducible ischaemia with stress echo or stress ECG testing, a battery of Nourin-dependent miRNAs were measured and compared to both healthy controls and a STEMI group. Two particular miRNAs, miR-137 and miR-106b-5p both showed marked elevation at baseline in those with positive stress tests, with >1,000- and 100-fold changes respectively (18). In the same study, levels of miR-137 were significantly higher in STEMI patients compared to the positive stress test group, suggesting capacity to stratify between normal, ischaemic, and infarcting myocardium. The role of these miRNAs in the heart is a matter of ongoing investigation, but there is established evidence for miR-137 as anti-inflammatory and anti-oxidant in neuronal tissue during ischaemic stroke (53).

#### In Summary: Future of Ischaemic Markers in Clinical Practice

The established inflammatory “prodrome” preceding acute myocardial events presents an opportunity to detect – and subsequently manage – myocardial ischaemic events in the earliest stages of injury (50). As this inflammatory picture shifts to mirror chronic disease processes, other markers such as phenylalanine and novel miRNAs above may provide prognostic value in the non-acute ischaemic heart.

### Biomarkers of Fibrosis and Post-infarct Remodeling

The heart's response to ischaemic insult through fibrosis and subsequent remodeling is a principal determinant of it's long-term function (54). The ability to stratify or predict this response prior to its development will become increasingly important in guiding management as promising anti-fibrotic cardiac therapies move closer to the clinical sphere.

#### The Osteopontin/Galectin-3 Axis

Osteopontin (OPN) is a is a glycoprotein expressed in various cardiac cells that may exist in either an “immobilized” state bound to the extracellular matrix, or as a soluble cytokine mediating inflammatory integrin activation (55). This ability to translocate may imply a messenger role in the remodeling response to physiological and pathological stimuli – accordingly, OPN has been found to be elevated in several animal models in response to biomechanical cardiac stress. As a biomarker, OPN showed diagnostic utility in a cohort of 420 HFrEF patients as well as a strong correlation with NYHA disease severity classification (19). Further, multivariate analyses in the same study found elevated levels to carry a hazard ratio of 2.3 for prediction of 4-year mortality even after accounting for clinical and biochemical features including NT-proBNP.

Osteopontin also triggers the secretion of Galectin-3 (Gal-3), a pro-fibrotic agent released from macrophages (56). In fact, Gal-3 has already seen recognition clinically by the FDA in the prognostication of chronic heart failure (57). Serial echocardiography in 240 advanced heart failure patients (NYHA 3/4) showed Gal-3 predicted change in LV end-diastolic volumes at 3 months compared to baseline, while no such similar significant trend was identified for NT-proBNP (20). Thus Gal-3 has a putative role in detection of both heart failure and acute myocardial infarction – the latter being more controversial in published literature (58).

#### Post-translational Modification of Troponins

High sensitivity cardiac troponin is one of the most widely used cardiac biomarkers in current clinical practice (59). Its role in diagnosis of myocardial infarction, myocarditis, and other acute cardiac events is well established. More recently, measurement of post-translation modification of troponins has been shown to reflect the degree of compromise in their function as contractile elements. In particular, phosphorylation of troponin T at serine Ser-207 has been shown to correlate with degree of left ventricular remodeling on echocardiography at 1 year follow up after myocardial infarction (22). Importantly, these blood biomarker changes were observable within the first week post-infarct, well before the echocardiographic changes that they predicted. A separate patent is held for nitrated cardiac troponin I, the result of reactive nitrogen species-driven post-translational modification which purportedly predicts myocardial ischaemia with or without infarction [patent US10175250B2]. It is reportedly detectable immediately after reperfusion of balloon-induced ischaemia in a pig model of left circumflex occlusion, however further publications on this beyond patent data are lacking.

#### Phenylalanine

The essential amino acid phenylalanine has been shown play a role in normal cardiac aging, cellular senescence, and fibrosis. Cell culture studies suggest a causative effect between phenylalanine and heart disease in mouse and human models through promoting aging-related redox and epigenetic changes (60). A recent human study in patients admitted to hospital with critical heart failure showed that elevated phenylalanine predicted 1 year all-cause mortality independent of several traditional risk factors (age, atrial fibrillation, cholesterol), mortality scores (SOFA, APACHE II), and inflammatory markers (CRP, transferrin, IL-8, IL-10) (21). Levels >112 μM carried a mortality hazard ratio of 5.06, and a hazard ratio of 2.57 after accounting for the above risk factors. Interestingly, inflammatory states have been shown to attenuate phenylalanine catabolism, suggesting a causative link between pro-inflammatory states and phenylalanine-mediated fibrotic risk (61).

#### In Summary: Future of Fibrosis Markers in Clinical Practice

Cardiac fibrosis is classically an irreversible step in the progression of disease with therapies only indirectly managing hemodynamics and fluid status (62). However emerging antifibrotic therapies suggest a growing role for quantifying and characterizing the degree of an individual's fibrotic state, potentially guiding therapy as well as providing prognostic value (63, 64).

### Biomarkers of Hemodynamics and Myocardial Contractility

Compromised myocardial contractility and/or relaxation is a hallmark of established cardiac disease (65). In fact, it is the sole finding in asymptomatic left ventricular dysfunction (ALVD), or “pre-heart failure,” a condition lacking appropriate screening tools and known to progress to true heart failure (66). ALVD has been found to have a prevalence of 6.8% in a random population of 75 year olds, and more commonly in men and those with coronary artery disease, hypertension, or ECG changes (67). With ALVD carrying a four-fold increase in mortality over 6 years, and cardiac contractility being a driving force behind heart failure symptoms particularly in HFrEF, quantification of contractility and the molecular mechanisms underpinning it are paramount.

#### Circular RNAs Including MICRA

Circular RNAs are particularly promising blood biomarkers due to their resistance to degradation compared to other elements of the blood transcriptome (68). Myocardial infarction-associated circular RNA (MICRA) is a circular RNA (circRNA) elevated during myocardial infarction that has shown particular prognostic value. The functional or pathophysiological role of MICRA remains to be fully elucidated, but experimental evidence is promising – in a cohort of 472 acute MI patients, MICRA levels at reperfusion correlated with ejection fraction at 4 months (23). A number of further circRNAs have been implicated in ischaemia/reperfusion, cardiac senescence, fibrosis, and remodeling, making for a promising area for future exploration (69).

#### Leukocyte-Derived Gene Biomarker Panel

ALVD is a recognized precursor to symptomatic heart failure, diagnosable through imaging studies such as *via* the application of echocardiography and tissue doppler techniques that are impractical to apply at an asymptomatic population screening level (70). A panel of seven genes characterized in peripheral blood may provide a biomarker signature of pre-clinical heart failure (24). Analysis of the leucocyte transcriptome in 294 patients without overt heart failure revealed that levels of FECH, TMEM79, FBXW7, NGFB, ALK, UBN1, and SLC43A2 in peripheral blood were able to predict ALVD with 87% accuracy and 100% precision. These genes encode for membrane proteins, mitochondrial components, and transcription factors not previously associated with heart disease prognosis. In contrast, NT-proBNP is useful in ruling out ALVD but shows poor positive predictive value (71).

#### Ang2 and TSP2

The protein angiopoietin-2 (Ang2) is a complex mediator of angiogenesis with seemingly mixed pro- and anti-angiogenic activity depending on its molecular context (72, 73). Ang2 also mediates pericyte detachment to alter vascular permeability in states of inflammation. Conversely, thrombospondin-2 (TSP2) is a potent anti-angiogenic factor with additional function in regulating myocardial matrix integrity (74, 75).

In a large population of 1,315 patients, proteomic analyses identified Ang2 and TSP2 as two particularly promising biomarker candidates across multiple cohorts. In dyspnoeic patients presenting to the emergency department, both were independently useful in distinguishing acute heart failure from alternative causes of dyspnoea after accounting for NT-proBNP level (25). Secondly, in a longitudinal study of 768 patients in the community without known heart disease, both markers predicted development of heart disease at 20 year follow up with hazard ratios of 1.36 (Ang2) and 1.29 (TSP2) per standard deviation of biomarker level, again independent of NT-proBNP (25). Interestingly, in the same study levels of both biomarkers declined significantly – by approximately 80% – in a third cohort of post-cardiac transplant patients; implying these are true biomarkers of local cardiac disease rather than surrogates reflective of systemic illness or indicative of comorbidities.

#### The GH/IGF-1/IGFBP2 Axis

Growth hormone (GH) and insulin-like growth factor 1 (IGF-1) are involved in intracellular calcium handling, myocardial contractile proteins, and systemic vascular function (76). IGF-1 binding protein 2 (IGFBP2) is a downstream regulator of IGF-1 at both the tissue and blood level, and in contrast to the more abundant IGFBP1, levels of IGFBP2 exhibit less post-prandial fluctuation (77). Analysis of plasma from 870 patients across multiple cohorts – two cohorts of outpatient chronic stable HF and 1 of patients presenting to the emergency department with acute decompensated HF – revealed positive associations with mortality, cardiac transplant, or need for LVAD (26). Importantly, a combined model of IGFBP2 and NT-proBNP produced stronger associations with these endpoints than using NT-proBNP alone. IGFBP2's role in CVD may be multifactorial, with associations also shown with intima-media thickness and myocardial infarction, while data on cardiometabolic endpoints such as obesity and metabolic syndrome paradoxically show negative correlations with IGFBP2 in literature (78–80).

#### In Summary: Future of Haemodynamic Markers in Clinical Practice

Taken together, these markers are promising as a means to assess ventricular function across a range of patient groups. The above trials demonstrate potential in asymptomatic, known heart failure, and post-infarct cohorts. Further exploration of these molecules may also provide mechanistic insights into phenotypes such as ALVD and HFpEF, which are poorly understood (81).

### Biomarkers of Metabolic Derangement and Mitochondrial Dysregulation

Metabolic syndrome is a key culprit in the modern-day heart disease phenotype (82). Diabetes, obesity, and subjective assessment of lifestyle factors capture only a subset of cardiometabolic disease and may be undetected before established cardiovascular damage. The intracellular manifestations of these changes center around mitochondrial dynamics, energy handling, and lipid modifications, and are promising candidates for metabolic biomarkers.

#### Plasma Lipidomics Panel

Lipids are implicated in atherosclerosis both locally in foam cells and the necrotic lipid core, and systemically as mediators of inflammation (83). In a cohort of 220 that compared healthy patients to those with stable and unstable angina, liquid-chromatography mass-spectrometry generated a panel of 105 lipids which improved predictive power in disease state classification when used alongside traditional risk factors (27). Sub-analysis of component lipids revealed that some may correlate with plaque burden, whilst others relate to plaque instability. For example, the lipid groups alkylphosphatidylethanolamine [PE(O)] and phosphatidylethanolamine plasmalogen [PE(P)] were only significant in the unstable CAD group with no difference seen between the control and stable CAD groups.

#### Mitochondrial ncRNAs Including LIPCAR

Mitochondria account for almost a third of the cardiomyocyte by volume – in keeping with their essential role in both cardiac health and disease (84). LIPCAR is a mitochondrial long non-coding RNA (lncRNA) shown to be elevated in chronic HFrEF, and is positively correlated with cardiovascular mortality in this cohort (28). Interestingly, LIPCAR levels were lower at baseline following infarction in patients who underwent more significant LV remodeling. This trend reversed in the following 1–12 month period, with increased LIPCAR in extensively-remodeled patients implying a dynamic role of LIPCAR in the natural history of cardiac remodeling. Non-coding mitochondrial microRNAs (mitomiRs) have also shown promise as biomarkers, possibly secondary to their post-transcriptional regulation of mitochondrial functions including apoptosis, calcium homeostasis, and energy metabolism (85, 86). Plasma profiling studies of 206 patients demonstrated 7 mitomiRs (miR-18a-5p, miR-26b-5p, miR-27a-3p, miR-30e-5p, miR-106a-5p, miR-199a-3p, and miR-652-3p) that both predicted 180-day mortality in heart failure, and differentiated acute HF from acute exacerbation of COPD (29).

#### Antibodies to MAA Adducts

Oxidative stress and lipid peroxidation have been implicated in atherogenesis in multiple studies by contributing to endothelial dysfunction and being a strong predictor of CAD events (87, 88). However, measurements of dysregulated oxidative signaling have been challenging. This has substantial implications for the translation of any novel antioxidant supplements or therapies (89), as an ideal clinical trial would be targeting individuals with high “oxidative stress,” and have a biomarker that could measure response to therapy. Biomarkers of dysregulated oxidative signaling may also have advantages for detection of early cardiometabolic disease given its integral role as a downstream mediator not only of many traditional risk factors but also of many emerging atypical drivers of disease (87). Malondialdehyde (MDA) is formed during lipid peroxidation and has been associated with endothelial dysfunction and CAD events (30, 90, 91). MDA breakdown can form malondialdehyde-acetaldehyde (MAA) adducts which contribute to atherogenesis through its proinflammatory, cytotoxic and potential protein modulating effect (30, 92). A cross-sectional study of 236 patients demonstrated that detection of different antibody isotypes to MAA adducts were associated with separate CAD states; for example, higher levels of anti-MAA IgM and IgG antibodies were associated with acute myocardial infarction, whilst CABG patients had comparatively higher anti-MAA IgA antibodies (30).

#### Homoarginine

Homoarginine is a nonproteinogenic amino acid that is suspected to have a protective cardiometabolic effect (31, 93, 94). The mechanism underlying this effect is unknown, although it is suspected that this association is driven by homoarginine-mediated nitric oxide production and subsequent protective vascular effects. Several studies have supported the potential the protective cardiometabolic effect of homoarginine through correlation (93), most notably in a cohort study of 3,305 patients finding low homoarginine levels were associated with increased cardiovascular and all-cause mortality (31). The study also demonstrated homoarginine levels were inversely related to markers of endothelial dysfunction (e.g., ICAM-1 and VCAM-1).

A more recent study of 2,106 patients reported several of the same correlations, however, when a Mendelian randomization approach was instituted, no significant causal associations of cardiometabolic or vascular protective effects were observed (95). Although the evidence surrounding homoarginine's role as a cardiac biomarker is debated (95), recent reviews suggest there may still be scope for its clinical use (93).

#### In Summary: Future of Metabolic Biomarkers in Clinical Practice

These markers stand to carry increasing importance given the growing recognition of multiple phenotypes within cardiometabolic disease (96). As such, characterizing the specific mechanisms involved in a given patient's disease alongside their genetic predispositions may become a larger part of clinical practice, prognostication, and management of CVD.

### Applications of Novel Biomarkers Across Different Clinical Settings

Biomarkers are applied across the full spectrum of clinical cardiovascular care. In the case of coronary artery disease and ischaemic heart disease, this extends from primary prevention to the diagnosis and risk assessment of acute coronary syndrome, to secondary prevention. Currently, screening in primary care is focused on traditional risk factors such as hypertension and dyslipidaemia, but there is an unmet need for markers that integrate these with the host response. Screening for risk factors and coronary artery disease may help target efforts in patient health education and behavior modification early in disease processes. In the setting of an acute coronary syndrome, some markers are focused on accurate diagnosis (97, 98). Currently, troponin, reflecting heart muscle damage, lacks the specificity to diagnose an atherosclerotic mechanism that would benefit from invasive angiography and percutaneous intervention, vs. potential mimic conditions such as Takotsubo cardiomyopathy or myocarditis. Improvements in single or combination biomarkers may help avoid unnecessary coronary angiography, particularly if combined with non-invasive imaging modalities (99). In general, for secondary prevention for IHD markers are currently similar to those used in primary prevention screening, although clinical action is more rigorous, with aggressive targets for pharmacological management of LDL cholesterol and BP in patients who have already suffered a major adverse cardiovascular event. Biomarkers may also be beneficial in tertiary prevention settings where the clinical application of said biomarker is related to therapeutic monitoring, such as in the case of BNP to monitor heart failure management (97, 98). Many of these scenarios open the opportunity for allied health professionals, such as clinical nurse consultants, to utilize biomarkers within the scope of their outpatient clinical practice, following guidelines for adjusting therapies and identifying warning signs.

## Biomarker Panels For Guiding Therapy

The classical “one size fits all” approach to CVD treatment based on early epidemiological data stands to be replaced by precision medicine, with therapies tailored to the individual patient (100). The growing field of pharmacogenomics emphasizes the need for therapy informed by both patient and disease factors, and with this comes the need to move toward utilizing integrated biomarker tools as adjuncts to clinical decision making. This section discusses biomarker panels which generate clinical scores to assist in developing personalized therapeutic regimens.

### Markers of Platelet Dysfunction May Be Used to Titrate Antiplatelet Therapy in Heart Disease

Where many major cardiac risk factors including diabetes, hypertension, and dyslipidaemia all have observable outcomes to guide treatment regimens, current standard of practice lacks the ability to practically measure case-by-case potency of antiplatelet therapy (101, 102). The epidemiology of clinically relevant resistance is controversial and poorly characterized but ‘laboratory' resistance has been found in meta-analyses to be present in 23%−40% of cases, based on tools such as light transmission aggregometry, impedance aggregometry, or platelet function analysers (103). Thus aspirin resistance often goes undetected, which is particularly concerning given its association with other comorbidities such as insulin resistance (104). A panel of 65 proteins or their associated RNAs measured in peripheral blood has been developed as an “aspirin response signature,” six of which were incorporated into a platelet function score reflecting response to aspirin (32). This signature was also correlated with adverse outcomes of myocardial infarction and death independent of other risk factors, implying identification of an aspirin-resistant population with a subsequent increase in adverse outcomes.

### Biomarker Panels to Guide Choice of Antihypertensive Medications in Heart Failure

Another panel proposes 16 biomarkers to guide the choice of antihypertensive pharmacotherapy in the context of heart failure. Several of these have already been shown to have prognostic value such as BNP, NT-proBNP, ST2, Gal-3 or cardiac troponins (105, 106). An individual patient data meta-analysis of 2,000 individuals found that heart failure therapy agent or dose guided by natriuretic peptide levels improved all-cause mortality in patients under 75 when compared to standard clinical care (107). Whilst the strength of this evidence is debated (105), the potential utility of multi-marker approaches is being explored to improve prognostic accuracy (108). For example, a cohort study of 195 patients found a combination of sST2 and BNP improved prognostic accuracy in HFrEF patients (109). The merits of using a multimarker approach thus appears sound as a method to guide the intensity of heart failure therapy (105).

Furthermore, there may be scope for this biomarker panel to guide the choice of pharmacotherapy. An analysis of 499 patients from the TIME-CHF study showed the relationship between different therapies and biomarkers (33). One of the significant findings included that high cystatin C levels were associated with improved outcomes (mortality and hospitalization) in patients treated with higher spironolactone doses. The combination of all these biomarkers as part of broader multimarker panel may present significant clinical utility in progressing personalized heart failure therapies.

### Inflammatory and Structural Proteins Predict Individual Response to Cardiac Resynchronisation Therapy

Cardiac resynchronisation therapy (CRT) – when successful – may improve quality of life, mortality, hospitalization, and NYHA class in select HF patients, and even partially reverses detrimental cardiac remodeling (110, 111). A number of trials have attempted to identify demographic factors that predict response to CRT, with modest success (112–114). A panel made up of 4 markers: soluble ST2 (sST2), soluble tumor necrosis factor receptor-II (sTNFr-II), matrix metalloproteinase-2 (MMP-2), and CRP have been combined into the Biomarker-CRT score to predict likelihood of a favorable response from peripheral blood (34). Just over half of study participants had a positive CRT response, while those with the lowest score of 0 were more than five times more likely to respond than those with the maximum score of 4. This effect was independent and additive to clinical and demographic factors.

### A Lipidomic Signature Can Predict Pravastatin Efficacy in Secondary Prevention

Lipid-lowering statin therapy is widely used in cardiovascular secondary prevention, making for one of the most widely prescribed drugs in the world (115). A lipidomic analysis of over 10,000 samples from 5,000 individual in the Long-Term Intervention with Pravastatin in Ischaemic Disease (LIPID) study, a large clinical trial of statin efficacy in secondary prevention, identified a two lipid signature, PI(36:2)/PC(38:4), that could stratify people into those who received a reduction in risk in response to statin treatment and those who did not (116). With this study also demonstrating as many as 25% of patients not receiving any measurable cardiovascular risk reduction from pravastatin therapy, these types of risk markers hold promise for a more personalized approach to risk stratification within the existing risk management framework.

## The Legal And Commercial Environment Surrounding Cardiovascular Biomarkers

Commercialisation pathways play a critical role in allowing for the rigorous translation of biomarkers or other medical innovations to the clinic (117). Ultimately, the pathway ensures clinical efficacy and utility, optimizes methods for measurement, defines standard operating protocols for the assay, identifies the market, and provides the service. Partnership between researchers in academia and industry is critical for the success of this translational pipeline. Traditionally patents were awarded for individual biomarkers and their application to diagnosis or risk prediction in particular conditions. However, increasingly patents covering the measurement of naturally occurring molecules have been challenged.

The ability to patent biomarkers and consequently enforce said patents is a complex issue that has undergone significant recent regulatory changes, and vary between countries and regulatory bodies. Notably in the United States, three key decisions in Mayo v Prometheus, AMP v Myriad Genetics and Alice Corp. v CLS Bank International placed significant restrictions on what biomarker claims could be patented (118).

- The Mayo decision of 2012 highlighted how natural processes (in this case being the measurement of a metabolite to titrate drug dose) cannot be patented (119)
- The Myriad decision of 2013 demonstrated that naturally occurring products (in this case the detection of DNA fragments) cannot be patented (120).
- The Alice decision of 2014 demonstrated that abstract ideas (in this case being the use of technology to exchange financial information) cannot be patented (121).

Thus, current and future claims will need to pass three tests to be patent eligible: first, that it differs from nature or natural processes; second, that it consists of inventive concepts (i.e., not abstract ideas or natural laws); and third, that it differs significantly from routine clinical practice (122). Despite these barriers, subsequent decisions demonstrate patents are still achievable, albeit with claims requiring increased specificity to be granted – for example, patents granted between 2014 and 2018 (the time since these landmark decisions) in personalized oncology diagnostics have increased (118). Interestingly, there has been a shift in the presentation of these patents with the majority of new claims in this field being tied to therapeutic methods (53%). The trend of expanding patents to also include changes to treatment algorithms rather than only including diagnostic biomarkers has been rising since 2012, reflecting the changing legal precedence, particularly after the Mayo decision and the resulting requirements put on prospective patent holders (118). This trend in patent claim construction is also demonstrated in the field of cardiac biomarkers as detailed in Table 1.

In addition to patents, there are alternative pathways for a novel marker being implemented in the clinic that include licensing of “know how” and platforms or tools to rigorously measure the marker and communicate results to physicians and patients. As detailed in Table 1, some of these measures utilized in the field of cardiac biomarkers include decision matrices (MMP-9, adiponectin), computerized biomarker risk algorithms (angiopoeitin-2, anti-hypertensive panel), means of monitoring therapeutic effectiveness (phenylalanine, MICRA, mitomiR panel, aspirin response signature) and small molecules implicated in cardiovascular pathophysiology (galectin-3, thrombospondin-2, homoarginine). However, more complex biomarker panels particularly in the fields of metabolomics and lipidomics where panels may contain dozens to hundreds of individual metabolites are likely to also depend heavily on “know how” of both the measurement and the algorithm, to drive the commercialization process (123). Whether or not the more complex algorithms themselves will represent a patentable form of intellectual property remains to be determined as this field develops over coming years. In either event, it will be important to develop a viable commercialization pathway for these developments if we are to maximize the health benefits from this technology.

Despite restrictions on patenting biological processes, there remain a number of pathways to commercially incentivise emerging biomarkers. Patents may still be taken out on inventions that utilize biomarkers to produce clinically relevant results, including assays. An example of this is the cardiac troponin – no single patent is held on its detection but a number of “high sensitivity” assays have been patented by separate groups over the years, resulting in increasing sensitivity through a competitive commercial environment while still providing financial incentive (124). This has implications not only in earlier detection of acute myocardial infarctions but also opens the door for its use in a wide range of diseases involving myocardial strain or insult including heart failure, pulmonary embolism, and sepsis (125).

Similarly, novel platforms for detection and quantification emphasizing rapid delivery of results are also grounds for patenting. Aptasensors are a form of biosensors using highly specific oligonucleotides or short peptides to bind to a target analyte and produce a signal through a transducer that can then be quantified (126). Aptasensors provide several advantages over classical antibody assays including a broader range of potential analytes, portability, and the potential for point-of-care testing designs (127). Again, troponins are a chief candidate for timely detection in point-of-care or out-of-hospital settings (128), and other markers such as BNP, IL-6, and CRP have also been had aptasensor patents developed [WO2017062349A1]. More conventional lateral flow assays have also shown promise in both qualitative and quantitative point-of-care applications (129).

### Challenges in Progressing From the Bench to the Bedside

Translation of biomarkers from the laboratory into the clinical sphere brings with it a number of technical challenges. The most obvious of these is validation of laboratory findings in relevant clinical cohorts in a reproducible manner, but there are several other hurdles to consider (130). Detection of a given marker should be timely and financially practical – this is particularly important in markers of ischaemia and infarction where there is a well-recognized correlation between time-to-treatment and clinical outcomes (131). To this end, the development of a routinely available assay that is feasible to use outside of a research setting requires significant assay optimisation, while a number of point-of-care technologies such as aptasensors and lateral flow tests show promise in rapid quantification of biomarkers including troponins (129, 132, 133).

Broadly speaking, a given biomarker should provide additional predictive value over established tests, typically requiring improved sensitivity and/or specificity to be demonstrated (134). However given the heterogenicity of pathophysiology in cardiac disease, markers that are less sensitive or specific than established markers may still complement them by providing information on different mechanisms of disease and prognosticate, stratify, or otherwise guide treatment in particular subgroups that are otherwise missed (135). Thus “panel” based markers provide an opportunity for combining biomarkers into a multi-mechanism approach. Beyond this, biomarkers guiding treatment necessitate not only improved predictive value but also a demonstration of improved outcomes for patients in a clinical setting. This is particularly challenging given the timeframes required to observe disease progression and long-term consequences of therapy. Researchers and research groups equipped with the skillset and resources for biomarker discovery and assay development may not be the same as those with access to clinical populations and driving therapeutic decision-making over time, thus leading to a point of fragmentation along this translational pipeline.

Finally, there is the hurdle of approval for clinical use by governing regulatory bodies. Indeed, of the biomarkers discussed in this paper only galectin-3, sST2, and hsCRP have recognized cardiovascular indications by the FDA despite the promising human clinical data presented above (58, 136, 137). This appears to be largely due to the lack of a clearly defined roadmap linking scientific, industrial, and regulatory bodies in a collaborative fashion (117).

## Conclusion

There remains a considerable unmet need for an improved armament of blood-based biomarkers in early detection and prevention of a variety of common cardiovascular disease states. Against this, hurdles to the development and commercialization of cardiovascular biomarkers remain. The markers presented above carry promising prognostic and predictive value that suggests applicability in guiding therapy, however longitudinal data demonstrating a reflection in improved patient outcomes over time remains limited and should be an area of future attention. Improved molecular phenotyping with more advanced blood-based biosignatures will likely permit enhanced stratification of patients in subgroups where distinct biology may be shared, and subsequently drive personalized treatment regimens. Further, the growing understanding of these disease phenotypes provided by emerging biomarkers may implicitly lead to the development of therapies specifically targeting these subgroups. This has the potential to assist in accelerating translation of biologically relevant therapies – such as novel small molecules and targeted RNA therapies – in groups enriched with relevant signaling abnormalities. Ongoing collaboration between academic and clinical leaders with industry and regulatory bodies are required to overcome hurdles in biomarker translation. Looking forward, exciting opportunities abound in this area that will enable the practice of cardiology to better embrace the precision medicine strategies of the future.

## Author Contributions

SS and JG drafted the initial version under the supervision of GF, with all other authors contributing additional sections and editing. All authors were significantly involved in the writing and editing of this manuscript.

## Conflict of Interest

The authors declare that the research was conducted in the absence of any commercial or financial relationships that could be construed as a potential conflict of interest.

## Publisher's Note

All claims expressed in this article are solely those of the authors and do not necessarily represent those of their affiliated organizations, or those of the publisher, the editors and the reviewers. Any product that may be evaluated in this article, or claim that may be made by its manufacturer, is not guaranteed or endorsed by the publisher.

## Abbreviations

ACS: acute coronary syndrome
ALK: anaplastic lymphoma receptor tyrosine kinase
APACHE II: acute physiology and chronic health evaluation II
Ang2: angiopoietin-2
ALVD: asymptomatic left ventricular dysfunction
BNP: brain natriuretic peptide
CABG: coronary artery bypass graft
CAD: coronary artery disease
circRNA: circular RNA
CRP: c-reactive protein
CRT: cardiac resynchronisation therapy
CVD: cardiovascular disease
ICAM: intercellular adhesion molecule
ICU: intensive care unit
IGF-1: insulin-like growth factor
IGFBP: IGF-1 binding protein
IgG: immunoglobulin G
IgM: immunoglobulin M
IL-8, IL-10, interleukin-8: interleukin 10
FBXW7: f-box and WD repeat domain containing 7
FECH: ferrochelatase
Gal-3: galectin-3
GH: growth hormone
HF: heart failure
HFrEF: heart failure with reduced ejection fraction
hsCRP: high sensitivity c-reactive protein
LIPCAR: long intergenic non-coding RNA predicting cardiac remodeling
lncRNA: long non-coding RNA
LOX-1: lectin-like oxLDL (oxidized low-density lipoprotein) receptor 1
LV: left ventricle
LVAD: left ventricular assist device
MAA: malondialdehyde-acetaldehyde adduct
MDA: malondialdehyde
MICRA: myocardial infarction-associated circular RNA
miR/miRNA: microRNA
mitomiR: mitochondrial miRNA
MMP: matrix metalloproteinase
ncRNA: non-coding RNA
NGFB: nerve growth factor (beta polypeptide)
NT-proBNP: N terminal-pro BNP
NYHA: New York Heart Association
OPN: osteopontin
PSGL-1: p-selectin glycoprotein ligand-1
RNA: ribonucleic acid
SLC43A2, solute carrier family 43: member 2
sLOX-1: soluble LOX-1
SOFA: sequential organ failure assessment
sST2: soluble suppression of tumorigenesis-2
STEMI: ST-elevation myocardial infarction
sTNFr-II: soluble tumor necrosis factor receptor-II
TMEM79: transmembrane protein 79
TSP2: thrombospondin-2
UBN1: ubinuclein 1
VCAM: vascular cell adhesion molecule
VEGF: vascular endothelial growth factor.
